# Current-Induced Thermal Tunneling Electroluminescence in a Single Highly Compensated Semiconductor Microrod

**DOI:** 10.1016/j.isci.2020.101210

**Published:** 2020-05-28

**Authors:** Cheng Xing, Wei Liu, Qiang Wang, Chunxiang Xu, Yinzhou Yan, Yijian Jiang

**Affiliations:** 1 Institute of Laser Engineering, Faculty of Materials and Manufacturing, Beijing University of Technology, Beijing 100124, China; 2 State Key Laboratory of Bioelectronics, School of Biological Science and Medical Engineering, Southeast University, Nanjing 210096, China; 3 Department of Materials Science and Engineering, Beijing Institute of Petrochemical Technology, Beijing 102617, China; 4 Key Laboratory of Trans-scale Laser Manufacturing Technology (Beijing University of Technology), Ministry of Education, Beijing 100124 China; 5 Beijing Engineering Research Center of Laser Technology, Beijing University of Technology, Beijing 100124, China; 6 Beijing Colleges and Universities Engineering Research Center of Advanced Laser Manufacturing, Beijing 100124, China

**Keywords:** Optical Materials, Devices, Materials Design

## Abstract

Here we demonstrate a novel and robust mechanism, termed as “current-induced Joule heating activated thermal tunneling excitation,” to achieve electroluminescence (EL) by the hot electron-hole-pair recombination in a single highly compensated semiconductor microrod. The radiative luminescence is electrically excited under ambient conditions. The current-induced Joule heating reduces the thermal tunneling excitation threshold of voltage down to 8 V and increases the EL efficiency ~4.4-fold at 723 K. We interpret this novel phenomenon by a thermal tunneling excitation model corrected by electric-induced Joule heating effect. The mechanism is confirmed via theoretical calculation and experimental demonstration, for the first time. The color-tunable EL emission is also achieved by regulation of donor concentration. This work opens up new opportunities for design of novel multi-color light-emitting devices by homogeneous defect-engineered semiconductors in future.

## Highlights

• Current-induced thermal tunneling EL is found in a homogeneous HC-ZnO microrod
• The high temperature is beneficial to the current-induced thermal tunneling EL
• The tunneling mechanism of Joule-heating-facilitated excitation is revealed
• The color-tunable EL emission is demonstrated by regulation of donor concentration

## Graphical Abstract

**Figure:**
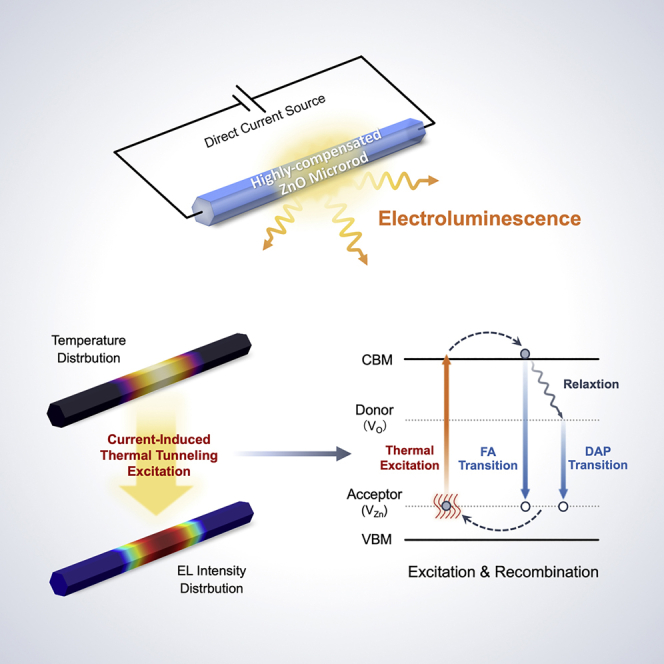

## Introduction

Intrinsic electroluminescence (EL), also called Destriau effect, was first observed in 1936 as ZnS:Cu phosphors were excited by a high electric field (Mach and Muller, 1982). It is the root of alternating current (AC) thin-film phosphor panel technology today, based on the impact excitation of deep/isolated defect centers, e.g., Cu, Mn, and Eu, (Ando and Ono, 1990, Howard et al., 1982, Que et al., 1998). It prevents the carrier loss at the junction interface due to the heterostructural barriers and surface states in carrier injection-based devices (Feng et al., 2019, Hao et al., 2018, Liu et al., 2018a, Ozgur et al., 2005, Wu et al., 2016). These deep luminescent centers dominated by short-range Coulomb potentials demonstrate quite different properties from the shallow donors and acceptors, e.g*.*, high stability of thermal ionization, multi-charge states, and large cross-sectional areas for carrier trapping (Grimmeiss, 1977, Kim et al., 2012, Meyer et al., 2005). However, the major drawback of EL devices is the quenching of luminescence at temperature greater than 400 K without thermal management, due to the increased non-radiative recombination rates (Willander et al., 2009). The microcavity and surface plasmon resonance structures have been found to be beneficial to enhance the luminescence for a potential approach for high brightness LEDs (Li et al., 2018, Liu et al., 2018b, Sun et al., 2020). However, a semiconductor with a high exciton binding energy is still the root to meet the thermal-quenching challenge, such as ZnO with an exciton binding energy of 60 meV (Ozgur et al., 2005). Although the electrically driven light emission was first demonstrated from a biased individual ZnO:Ga microwire in 2017 (Jiang et al., 2017), the intrinsic EL mechanism is unrevealed so far, especially the dual-contribution of Joule heating and impact excitation governed by the complex defect impurities, e.g*.*, Ga, Sb (He et al., 2017a, He et al., 2017b, Jiang et al., 2017, Jiang et al., 2019, Liu et al., 2017, Liu et al., 2018b, Sun et al., 2020).

Our previous work has demonstrated the stable acceptor-rich ZnO (A-ZnO) microrods/tubes grown by temperature-gradient-free optical vapor supersaturation precipitation (OVSP) (Hu et al., 2018a, Hu et al., 2018b, Huang et al., 2019, Wang et al., 2016, Wang et al., 2017a, Wang et al., 2017b, Wang et al., 2019b). The Zn vacancy (V_Zn_) acted as an acceptor level, activating donor-acceptor-pair (DAP) recombination with intrinsic compensation donors, i.e., oxygen vacancy (V_O_), for visible photoluminescence (PL) emission up to 773 K (Wang et al., 2017a). It forms a robust and generic luminescent center in wide band-gap semiconductors for high-temperature EL emission. Meanwhile, the strong self-compensation effect in A-ZnO reduces the free-carrier concentration and increases the electrical resistivity, resulting in significant Joule heating under a bias current (Fan et al., 2013, Wang et al., 2016). The highly compensated wide-bandgap semiconductor thereby provides a novel platform to investigate the high-temperature electron-hole-pair-related EL process for novel luminescent applications.

In this work, we report the first observation and validation of the current-induced Joule heating activated thermal tunneling excitation for EL emission from hot electron-hole-pair recombination in a highly compensated ZnO (HC-ZnO) microrod. The EL emission was observed at the center of a biased HC-ZnO microrod. The voltage threshold was reduced with the temperature increasing and the external quantum efficiency was boosted dramatically. It is the strong evidence on thermal tunneling excitation of hot hole and electron carriers for EL emission. This fundamentally differs from the incandescent light mechanism in previous studies (He et al., 2017a, He et al., 2017b, Jiang et al., 2017, Jiang et al., 2019, Liu et al., 2017). In order to validate the contribution of electron-hole-pair recombination in EL emission, the oxygen pressure was regulated in growth of HC-ZnO microrods for different V_O_-related donor concentrations. The influence of V_O_-related donor concentration on EL spectrum was understood. A theoretical model was also developed to estimate the recombination rates between conduction band minimum (CBM) and defect levels in the HC-ZnO microrod. Such a study opens up new opportunities to design novel wide-bandgap semiconductor EL devices with a simple structure for high-efficiency light emission at high temperature.

## Results

### Fabrication and Characterization of HC-ZnO Microrods

**Figure 1:**
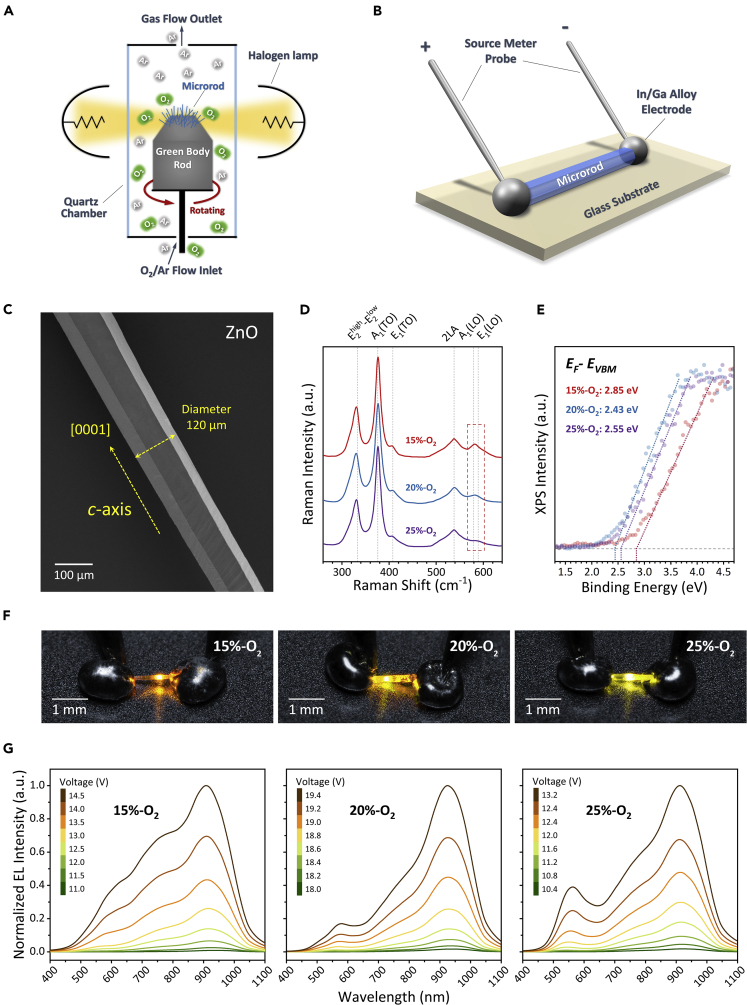
Fabrication and Characterization of HC-ZnO Microrods (A) Experimental setup of HC-ZnO microrods grown by OVSP. (B) Schematic of the MSM setup for an individual HC-ZnO microrod as an EL device. (C) Typical SEM image of an HC-ZnO microrod. (D and E) (D) Raman spectra and (E) XPS valence band spectra of HC-ZnO microrods grown in different oxygen pressures. (F) Optical images of EL emission from HC-ZnO microrods with different V_O_. (G) Evolution of EL spectra with applied bias voltages.

Figure 1A shows the setup for growth of HC-ZnO microrods with massive V_Zn_-related acceptors and Vo-related donors, as proposed in our previous studies (Hu et al., 2018b, Wang et al., 2016, Wang et al., 2017a, Wang et al., 2017b). To efficiently control the V_O_ concentration, the O_2_/Ar mixed carrier gas with variable oxygen pressure ratios (i.e., 15%, 20%, and 25%) was used during OVSP growth; a mixture of ZnO and graphite powders with a definite weight ratio of 2:1 was pressed as the reactive green body rod. Figure 1B demonstrates the metal-semiconductor-metal (MSM) setup for the EL emission from an individual HC-ZnO microrod. The microrod was placed on a cleaned glass substrate and coated with In/Ga alloy electrodes at both ends for Ohmic contacts (Wang et al., 2016). The bias voltages were applied on the microrod and the corresponding currents were measured via a source meter.

Figure 1C shows the typical morphology of an HC-ZnO microrod, of which the diameter was ~120 μm and the length was up to 1.5 mm. The hexagonal geometry is attributed to the wurtzite-type structure along the *c*-axis, i.e., [0001] direction (Wang et al., 2016). The Raman spectra were acquired by a high-resolution spectrometer, as shown in Figure 1D. The observation of longitudinal optical phonon (LO) modes with A_1_ and E_1_ symmetries at 576 and 589 cm^−1^ with the oxygen pressure reducing confirmed the increase of V_O_ concentration, owing to the low formation energy of V_O_ determined by first-principle calculation (Janotti and Van de Walle, 2007) and Fröhlich interaction (Alaria et al., 2006, Cheng et al., 2010). It should be noted that the inactive *E*^2^_high_ Raman mode at 437 cm^−1^ is attributed to the polarization of excitation laser parallel to the *c*-axis of the HC-ZnO microrod (Cusco et al., 2007). In Figure 1E, the XPS valence band spectra fitted by tangent lines (dotted lines) determined the energy differences between the Fermi level and valence band maximum (VBM), i.e., *E*_*F*_-*E*_*VBM*_, for the HC-ZnO microrods grown in 15%-O_2_, 20%-O_2_, and 25%-O_2_ corresponding to 2.85, 2.43, and 2.55 eV, respectively. It indicated the *n*-type of the HC-ZnO microrods fabricated in this work.

Figure 1F shows the EL emission with varied colors near the centers of microrods when the bias voltage was greater than a threshold for each sample with the specific V_O_ concentrations. This phenomenon is similar to the previous studies on incandescent light mechanism analog to the “hot spot” of tungsten filament (He et al., 2017a, He et al., 2017b, Jiang et al., 2017, Liu et al., 2017, Luo et al., 2019). The corresponding EL spectra with the increase of bias voltage are shown in Figure 1G. The emission covered the spectrum from visible to near-infrared bands in the range of 400–1,100 nm, composing of several emission peaks, rather than blackbody radiation with a broad peak (Luo et al., 2019).

### Temperature and EL Characteristics of HC-ZnO Microrods

The classic theory indicates two channels for EL emission from semiconductors: (1) carrier injection from electrodes, e.g., *p-n* and Schottky junctions; (2) electron-hole-pair generation by impact excitation/ionization of defect-bound or valence electron under a high electric field, e.g., metal-insulator-semiconductor (MIS) junctions (Zhang et al., 2016). However, the threshold of electric field intensity ~10^2^ V/cm significantly lower than the theoretical value (>10^4^ V/cm [Lehmann, 1982]) for impact excitation/ionization promoted a distinctive mechanism of EL emission in the HC-ZnO microrods.

**Figure 2:**
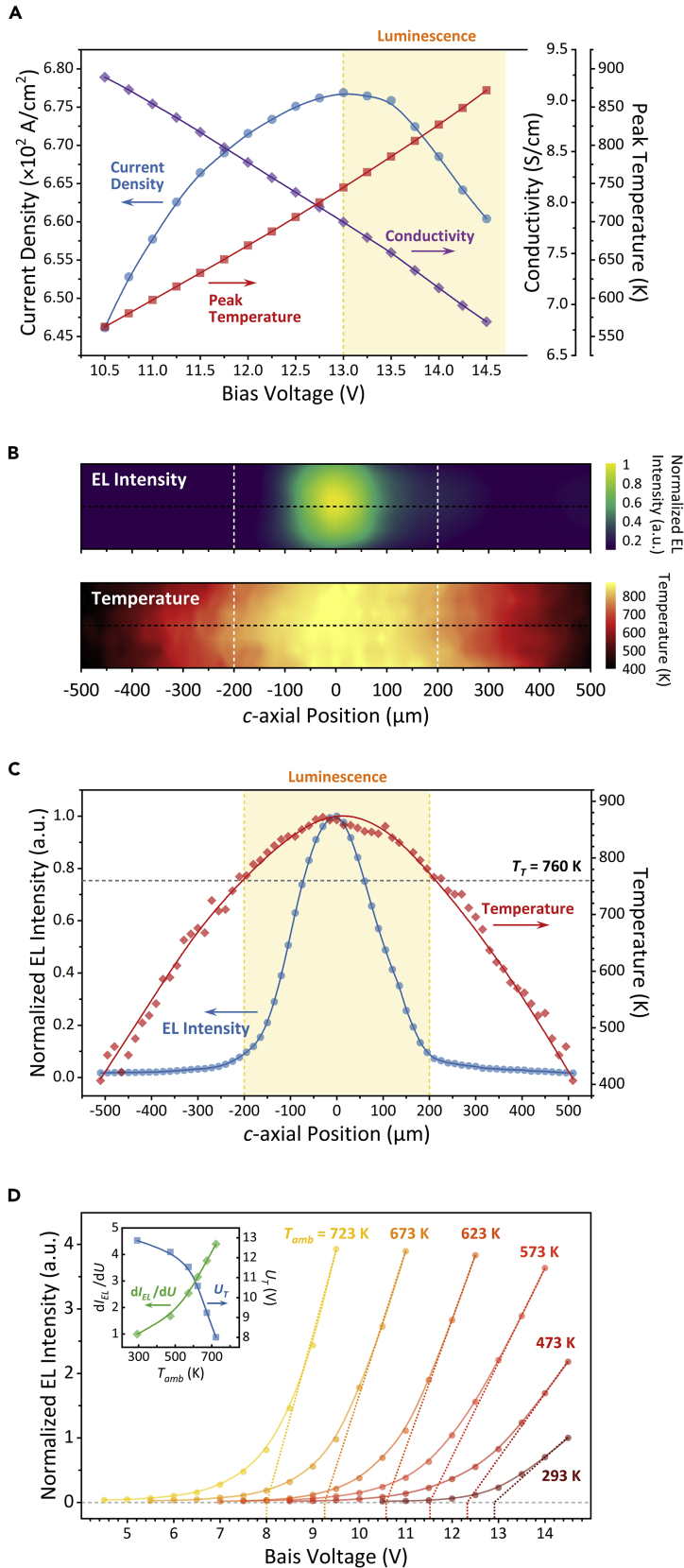
EL Characteristics of an HC-ZnO Microrod Grown in 15%-O_2_ Condition (A) Evolution of current density, conductivity, and peak temperature with bias voltage in EL mission. (B) 2D distributions of EL intensity and temperature of the microrod biased by 14.5 V. (C) 1D distributions of EL intensity and temperature on the *c*-axis of HC-ZnO microrod shown as the black dotted line in (B). (D) Effect of bias voltage on EL intensity under different ambient temperatures (*T*_*amb*_), where the inset illustrates the effects of *T*_*amb*_ on differential of EL intensity with bias voltage (d*I*_*EL*_/d*U*) and voltage threshold (*U*_*T*_) for EL mission.

Considering the thermal effects on excitation process (He et al., 2017a, He et al., 2017b, Jiang et al., 2017), the evolution of current density (*J*), conductivity (*σ*), and peak lattice temperature (*T*_*p*_) of the 15%-O_2_ HC-ZnO microrod with increased bias voltage were measured, as shown in Figure 2A. The low conductivity, similar to the undoped ZnO single crystal, confirmed the compensation effect occurring (McCluskey and Jokela, 2009). The increase of *T*_*p*_ with the bias voltage increasing was attributed to the current Joule heating. The continuous reduction of *σ* indicated the conductive property of the HC-ZnO is similar to metal materials, of which the electron mobility is reduced owing to the electron collision scattering with lattices and defects. The negative differential resistance (NDR) effect (d*J*/d*V*<0) with the bias voltage greater than 13.0 V indicated the metal-like electro-thermal transition in the HC-ZnO microrod (Gibson, 2018, Rashidi et al., 2016, Wang et al., 2019a). The lattice temperature caused by Joule heating is therefore described as (Zhao et al., 2011):

(Equation 1) $$T_{p}=T_{amb}exp\left(\frac{\alpha \sigma U^{2}}{8}\right)\;$$

where *T*_*p*_ is the peak temperature at EL emission zone, *T*_*amb*_ is the ambient temperature, *U* is the bias voltage, and *α* is the constant related to thermal conductivity *κ* and *T*_*p*_, where *α =*1*/κT*_*p*_. *κ* of 15%-O_2_ ZnO microrod at room temperature calculated by Equation (1) was ~79 W/m·K, lower than the ZnO single crystal with few defects (~100 W/m·K [Alvarez-Quintana et al., 2010]). It is due to the reduction of mean free path for phonon scattering resulting from the high concentration of defect in HC-ZnO (Asheghi et al., 2002).

To acquire the relationship of emission intensity and lattice temperature, the micro-EL/Raman spectra of the biased ZnO microrod were captured by 2D point-to-point spectral mapping with a spatial step of 15 μm. The lattice temperature was estimated by the peak shift of E^2^_high_ Raman active mode under the polarization of excitation laser perpendicular to the *c*-axis of the microrod (see Methods). The 2D distributions of EL intensity and lattice temperature during a 15%-O_2_ ZnO microrod biased by 14.5 V are plotted in Figure 2B. It can be clearly seen that the EL intensity was strongly dependent upon the lattice temperature. The EL region was located in the range of ~200 μm from the microrod center as indicated by the white dashed lines. Figure 2C further demonstrates the EL intensity and lattice temperature distributions on the *c*-axis of the HC-ZnO microrod. The peak temperature at the microrod center was ~872 K with bright light emission, of which the temperature was significantly higher than the dark zone around the electrodes of ~400 K. The temperature of 760 K as the dashed line indicated in Figure 2C was the temperature threshold, *T*_*T*_, for EL emission.

According to Wien's displacement law (Das, 2015), the temperature of blackbody radiation at 900 nm, i.e., the wavelength of EL intensity peak as shown in Figure 1E, should be ~3,200 K. It is significantly higher than the decomposition temperature of wurtzite-type ZnO at atmospheric pressure (~1,700 K [Kong et al., 2004]) and the measured temperature in Figure 2C. Figure 2D shows the bias-voltage-regulated EL emission at the ambient temperature *T*_*amb*_ ranging from 293 to 723 K. The elevated *T*_*amb*_ reduced the voltage threshold (marked by the dashed lines) for bright EL emission from 12.9 V at 273 K down to 8.0 V at 723 K. The corresponding differential of EL intensity with respect to bias voltage, *dI*_*EL*_*/dU*, was promoted by ~4.4-fold, indicating the luminescent efficiency was boosted. Therefore, the temperature-dominated EL channel for hot hole and electron carrier excitation and radiative recombination should be considered.

### Mechanism of Current-Induced Thermal Tunneling EL from HC-ZnO Microrods

**Figure 3:**
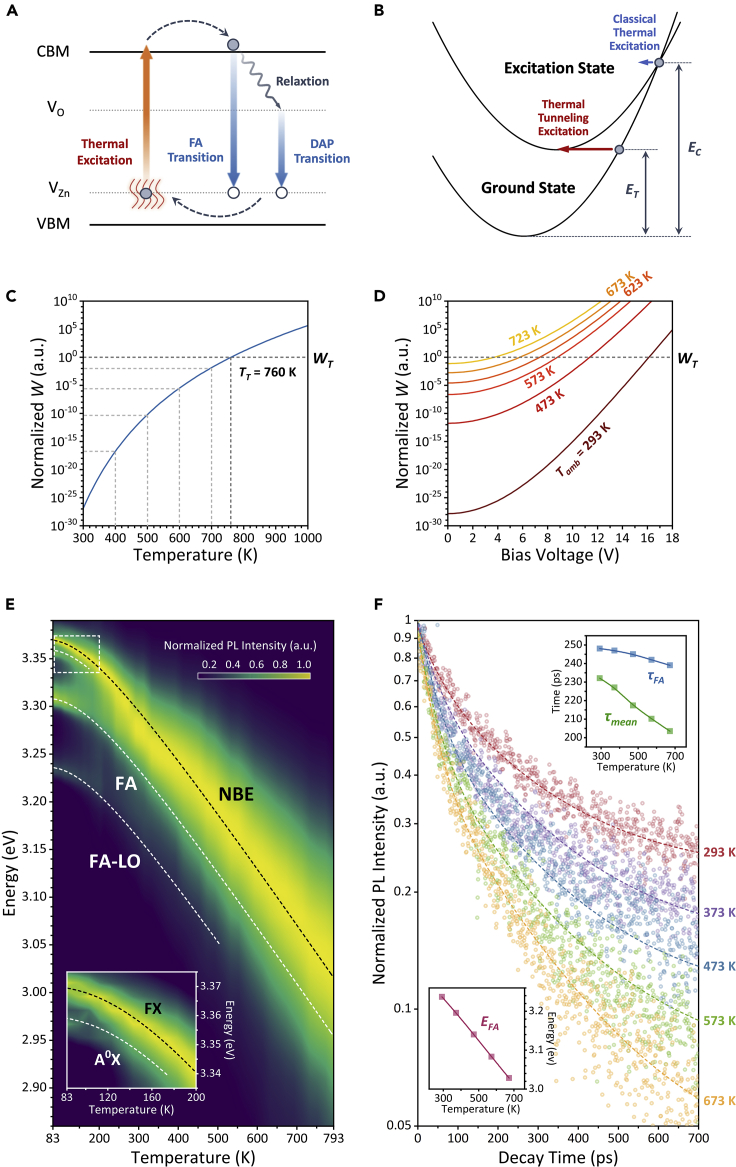
Mechanism of Current-Induced Thermal Tunneling EL (A) Schematic of excitation and recombination for EL emission in an HC-ZnO microrod. (B) Schematic of classic and thermal tunneling excitation channels. (C and D) Theoretical excitation probability of thermal tunneling excitation with (C) temperature and with (D) bias voltage at selected ambient temperatures. (E) Temperature-dependent PL spectra of an HC-ZnO microrod grown in 15%-O_2_, where the inset is the close-up view of the high-energy exciton recombination at the low temperature. (F) Temperature-dependent time-resolved PL spectra of the 15%-O_2_ HC-ZnO microrod. The dotted lines are the fitted decay curves based on the decay intensities (translucent point). The two insets exhibit the evolutions of fitted *τ*_*FA*_, *τ*_*mean*_, and *E*_*FA*_ with temperature.

The EL dynamic process of HC-ZnO was proposed in Figure 3A. The hot electron-hole-pair excitation and radiative recombination process includes that (1) current-induced Joule heating increases the lattice temperature forming a “hot spot” near the microrod center, where the thermal field activated the bound electrons in V_Zn_ into the conduct band (CB); (2) the excited electron-hole pairs were recombined by free-electron-to-neutral-acceptor (FA) transition as well as DAP transition by relaxation of hot electrons to V_O_ and then to V_Zn_-related holes.

The thermal excitation in this work was regarded as a multi-phonon process, where the transition of electron was from V_Zn_ (ground state) to CB (excitation state), as shown in Figure 3B. The tunneling excitation occurred when the thermal energy slightly exceeded the thermal binding energy from V_Zn_ to CB, *E*_*T*_, rather than a classical thermal excitation requiring energy *E*_*C*_ (Yassievich, 1994). The excitation tunneling probability, *W*, according to Huang-Rhys model is determined by (Karpus and Perel, 1986)

(Equation 2) $$W∼\;\;exp\left\{\frac{E_{T}}{\hbar \omega }\left[1-\frac{\beta }{4}-ln\left(\frac{4}{\beta }\left(exp\frac{\hbar \omega }{k_{B}T}-1\right)\right)\right]\right\}$$

where *β* = 0.26 is a constant*, ℏω=* 72 meV is the phonon energy, and *k*_*B*_ is the Boltzmann constant. Figure 3C shows the evolution of *W* with temperature elevation, where the probability threshold for EL emission, *W*_*T*_, corresponds to the excitation temperature threshold of *T*_*T*_ = 760 K in Figure 2C. Further considering the co-effect of ambient temperature and current-induced Joule heating (see Equation S4), the theoretical bias voltage threshold was reduced from 16.1 V at 293 K down to 3.8 V at 723 K as Figure 3D, showing good agreement with experiments and demonstrating the high-temperature preference of the HC-ZnO for EL emission.

Our previous work has confirmed V_Zn_ acting as stable recombination centers up to 773 K (Wang et al., 2016, Wang et al., 2017a). Figure 3E shows the temperature-dependent PL spectra of a 15%-O_2_ HC-ZnO microrod measured by a CW 325-nm-line He-Cd laser with the polarization perpendicular to the *c*-axis. The elevated temperature caused strong electron-phonon interaction to narrow the optical band gap as (Odonnell and Chen, 1991)

(Equation 3) $$E_{g}\left(T\right)=E_{g}\left(0\right)-S\left\langle \hbar \omega \right\rangle \left[\coth\left(\frac{\left\langle \hbar \omega \right\rangle }{2k_{B}T}\right)-1\right]$$

where *E*_*g*_(0) is the band gap energy at 0 K, *S* is the coupling constant, and ⟨*ℏω*⟩ is the mean phonon energy related to the strength of energy loss via phonon. The fitted curve, plotted as the black dotted line as shown in Figure 3A, yielded *E*_*g*_(0) = 3.37 eV and ⟨*ℏω*⟩ = 40.8 meV. The mean phonon energy is significantly higher than the standard ZnO single crystal (~12 meV [Choi and Ma, 2006]) owing to the high concentration of defects in the HC-ZnO.

The sharp peaks near 3.37 and 3.36 eV at 83 K are attributed to the free-exciton (FX) and neutral-acceptor-bound-exciton (A^0^X) transition, respectively (Alawadhi et al., 2007). According to our previous studies, the A^0^X transition was originated from the V_Zn_ acceptor level (Wang et al., 2016). The transition of FA located at 3.31 eV and the longitudinal optical phono replicas (FA-LO) with an energy separation of ~72 meV were observed in a wide temperature range from 83 to 793 K and 83 to 505 K, respectively. It indicates that the FA recombination rate related to V_Zn_ is comparable with NBE transition providing an EL emission channel.

The temperature-dependent time-resolved photoluminescence (TD-TRPL) spectra were acquired from 293 to 673 K to study the recombination efficiency related to V_Zn_ at high temperature. Figure 3F shows the decay of FA recombination at different temperatures of the 15%-O_2_ HC-ZnO microrod. The redshift of FA transition with the temperature elevation was corrected, as shown in the bottom inset. Considering the co-existence of FA and NBE transition, a double-exponential function was used to fit the decay curve as following (Lu et al., 2017):

(Equation 4) $$I_{L}\left(t\right)=A_{NBE}\;exp\left(\frac{-t}{\tau_{NBE}}\right)+A_{FA}\;exp\left(\frac{-t}{\tau_{FA}}\right)$$

where *I*_*L*_ is the luminescence intensity, *t* is the time, *τ*_*NBE*_ and *τ*_*FA*_ are the lifetimes of NBE and FA recombination, and *A*_*NBE*_ and *A*_*FA*_ are the proportional coefficients, respectively. The mean luminescence lifetime *τ*_*mean*_ (*=*(*A*_*NBE*_×*τ*_*NBE*_^*2*^*+ A*_*FA*_×*τ*_*FA*_^*2*^)/(*A*_*NBE*_×*τ*_*NBE*_ *+ A*_*FA*_×*τ*_*FA*_)) and *τ*_*FA*_ are shown in the top inset of Figure 3F. The reduction of *τ*_*mean*_ from 232 to 203 ps with the temperature increasing is attributed to the aggravation of non-radiative transition in NBE recombination, whereas *τ*_*FA*_ is decreased from 248 ps at 293 K to 239 ps at 673 K, indicating a high thermal stability for FA recombination. The TD-TRPL spectra confirmed the V_Zn_-related defects acting as an efficient recombination centers for EL emission.

### Mechanism of Color-Tunable EL Emission from HC-ZnO Microrods

**Figure 4:**
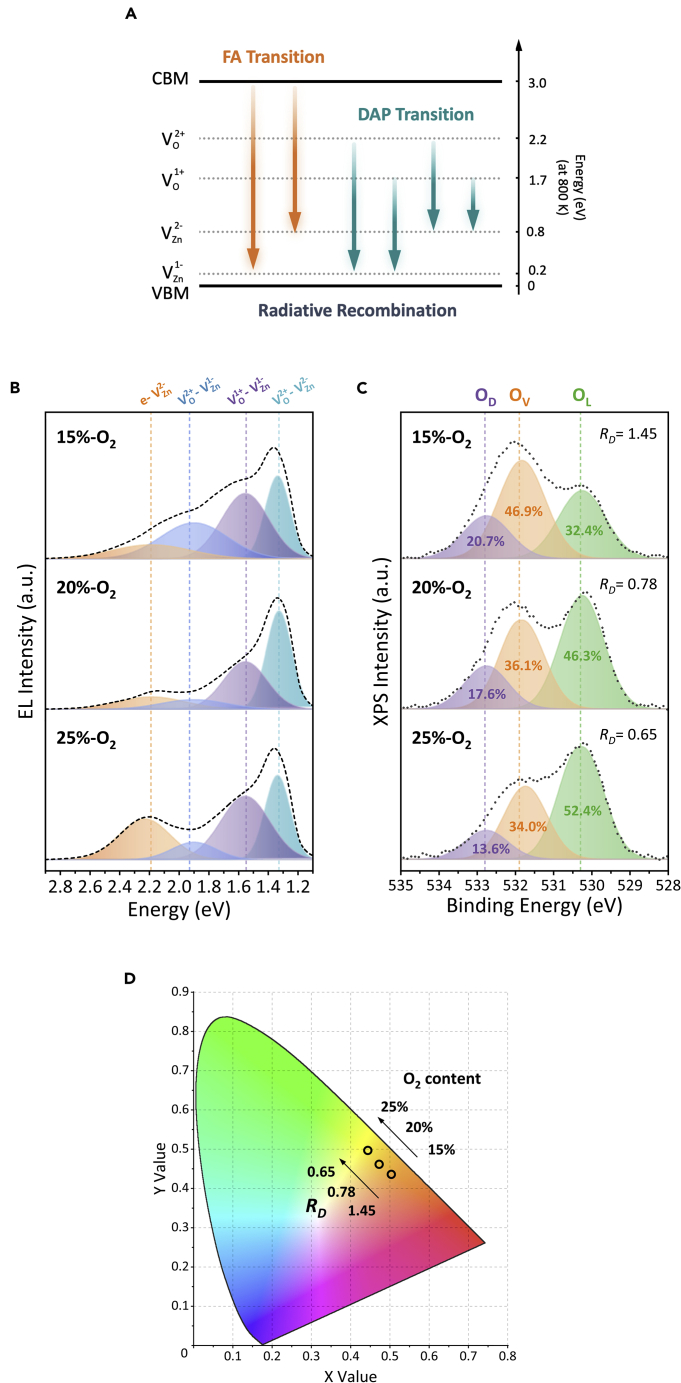
Origination of Color-Tunable EL Emission from HC-ZnO Microrods (A) Diagram of radiative recombination channels between fine energy levels. (B) EL spectra fitted by FA and DAP-related emission peaks for HC-ZnO microrods with different V_O_ concentrations. (C) XPS O1*s* spectra of HC-ZnO microrods. (D) Chromaticity distribution of EL emission from different HC-ZnO microrods.

In general, V_Zn_ composed of two fine levels, i.e., 0/1- (V^1-^_Zn_) and 1-/2- (V^2-^_Zn_), with 0.1–0.2 and 0.8–1.2 eV above the VBM (McCluskey and Jokela, 2009). The V_O_ also possessed 0/1+ (V^1+^_O_) and 1+/2+ (V^2+^_O_) with ~1.82 and ~2.51 eV above the VBM, respectively (Janotti and Van de Walle, 2007). In consideration of the bandgap narrowing at the high temperature, the diagram of corrected fine energy level and the radiative recombination channels are illustrated in Figure 4A. It can be seen that there are two and four potential channels of radiative recombination for FA and DAP transition, respectively, demonstrating different photon energies in EL emission.

To reveal the effect of V_Zn_- and V_O_-related defect concentrations on EL emission color, the EL emission spectra were fitted by Gaussian multi-peaks, as shown in Figure 4B. The EL emission peaks at 1.33, 1.55, and 1.92 eV originated from DAP transition of V^2+^_O_-V^2-^_Zn_, V^1+^_O_-V^1-^_Zn_, and V^2+^_O_-V^1-^_Zn_, respectively, contributing the most of EL emission energy. The narrow FWHM in the range of 0.1–0.2 eV indicated the intra-band transition caused radiation. The emission peak at 2.19 eV was attributed to the FA transition from the CBM to V^2-^_Zn_ (*e*-V^2-^_Zn_). The high energy of hot electrons in the CB caused the FWHM of emission peak up to 0.4 eV. The missed V^1-^_Zn_-V^2-^_Zn_ transition was due to the cutoff detection energy of the spectrometer (1.0 eV). The FA transition from the CBM to V^1-^_Zn_ (*e*-V^1-^_Zn_) was suppressed owing to the high binding energy of V^1-^_Zn_ for DAP recombination (Wang et al., 2016, Wang et al., 2017a).

According to the transition rate of equation between the energy levels (see Figure S1) and regardless of the non-radiative recombination, the emission ratio of DAP to FA transition (see Equations S5 and S6) is described as

(Equation 5) $$\frac{I_{DAP}}{I_{FA}}\propto \frac{\kappa_{DA}n_{D}}{\kappa_{eA}n}=\frac{\kappa_{eD}n}{\kappa_{eA}n}=\frac{\kappa_{eD}}{\kappa_{eA}}$$

where *I*_*DAP*_ and *I*_*FA*_ are emission intensities from DAP and FA transitions; *κ*_*DA*_ and *κ*_*eA*_ are the rates of DAP and FA transitions; *κ*_*eD*_ is the relaxation rate of hot electrons at the CB captured by V_O_; *n* and *n*_*D*_ are the concentrations of free-electrons at the CB and donor level of V_O_, respectively. Equation (5) indicated that the DAP radiative recombination was proportional to *κ*_*eD*_ determined by (Milnes, 1973)

(Equation 6) $$\kappa_{eD}\propto \sigma_{eD}n_{D}v$$

where *σ*_*eD*_ is the relaxation cross-section dominated by direction of electron movement and electric field strength and *v* is the thermal velocity of hot electrons following *v=*(2*k*_*B*_*T/m^∗^*)^1/2^, in which *m^∗^* is effective mass of electrons. Assuming the hot electrons moving backward to the applied electric field vector as well as the similar bias voltage and lattice temperature at the “hot spot” of the HC-ZnO microrod, the DAP recombination rate was hence strongly dependent upon the concentration of V_O_, i.e.*, n*_*D*_.

To accurately determine the concentration of V_O_, the equivalent parameter (*R*_*D*_) was proposed to represent the concertation of V_O_. Figure 4C shows the XPS spectra of O1*s* fitted by three Gaussian-Lorentzian peaks with the central energy of 530.3, 531.6, and 532.8 eV attributing to lattice oxygen (O_L_), oxygen vacancy (O_V_), and surface oxygen absorption (O_D_), respectively (Chen et al., 2000, Lu et al., 2016, Tay et al., 2006). *R*_*D*_ was estimated by the peak area ratio of O_V_ to O_L_ to determine the concentration of V_O_. It can be seen that *R*_*D*_ was decreased with the O_2_ content increasing during growth. It is in good agreement with the Raman results shown in Figure 1D. The capability of V_O_ concentration to regulate the EL emission color was therefore validated. Figure 4D shows the emission chromaticity values in the CIE color space by the EL spectra (Wang et al., 2016, Wang et al., 2017a, Yang et al., 2019). The emission color was tunable from orange (0.50, 0.44) to bright yellow (0.44, 0.50) with the decrease of *R*_*D*_ from 1.45 to 0.65. i.e.*,* the increase of O_2_ content from 15% to 25% in growth.

## Discussion

Evidence on current-induced Joule heating activated thermal tunneling excitation and hot electron-hole-pair recombination for EL emission in a highly compensated semiconductor microrod is presented in this work. It is confirmed that the intensity of EL emission is dependent upon the lattice temperature. The thermal tunneling threshold of voltage was down to 8 V with 4.4-fold luminescent efficiency promotion under the temperature up to 723 K. The color-tunable EL emission is also achieved by regulation of V_O_-related donor concentration. The hot electron-hole-pair radiative recombination opens up opportunities for defect-engineered wide-bandgap semiconductors as efficient light-emitting devices at high temperature.

### Limitation of the Study

We have developed a mechanism named current-induced Joule heating activated thermal tunneling excitation to achieve EL by the hot electron-hole-pair recombination in a single highly compensated semiconductor microrod. The element doping could future regulate the EL emission with a wider color range with a higher efficiency. The highly compensated doped-ZnO microrod is worthy of study. In addition, the structure of highly compensated semiconductors is limited in microrod by OVSP growth. The other structures, such as films, bulks, and nanowires, might be fabricated for industrial applications in the future.

### Resources Availability

#### Lead Contact

Further information and requests for resources and reagents should be directed to and will be fulfilled by the Lead Contact, Yinzhou Yan (yyan@bjut.edu.cn)

#### Materials Availability

This study did not generate new unique reagents.

#### Data and Code Availability

All data are available from the corresponding author upon reasonable request.

## Methods

All methods can be found in the accompanying Transparent Methods supplemental file.

## Supplemental Information

Document S1. Transparent Methods
