# Supplementary material for: Current-Induced Thermal Tunneling Electroluminescence in a Single Highly Compensated Semiconductor Microrod
Source: iScience. 2020 May 28;23(6):101210. doi: 10.1016/j.isci.2020.101210 (PMC7289767; doi:10.1016/j.isci.2020.101210)
Supplement: Document S1. Transparent Methods [file mmc1.pdf]

iScience, Volume *23*

Supplemental Information

Current-Induced Thermal Tunneling Electroluminescence in a Single Highly Compensated Semiconductor Microrod

Cheng Xing, Wei Liu, Qiang Wang, Chunxiang Xu, Yinzhou Yan, and Yijian Jiang

TRANSPARENT METHODS

Fabrication of HC-ZnO microrods. The HC-ZnO green body rod was synthesized by 99.99% ZnO (Alfa Aesar) and graphite powders with a definite weight ratio of 2:1. The mixture powder was pressed into a rod by isostatic pressing with 70 MPa for 30 mins. The dimensions of the green body rod were typically 6 mm in diameter and 10 mm in length. The optical vapor supersaturation precipitation (OVSP) was performed in an optical image furnace (Crystal Systems Co., Ltd. 10000H–HR-I-VPO-PC) equipped with four halogen lamps. The power of each lamp was first increased to 600 W for ~1000 °C on the top of green body rod within 10 mins. Then the lamp power was hold for 5 mins for OVSP process. Finally, the power was reduced to zero within 10 mins to relax the residual thermal stress. The green body rod was rotated with 10 rpm and the mixed carrier gas (O_2/Ar) was pumped into the chamber with 200 mL/min during growth.

Characterization of electrical properties. An individual microrod was placed onto a silica substrate. The In/Ga electrodes was deposited onto both ends of the microrod for ohmic contact. The bias voltage *U* and current *I* were applied and measured by a source meter (Keithley SMU2600B). The morphology of HC-ZnO microrod was simplified into a hexagonal prism in calculation of electric parameters. The current density *J* and conductivity σ are therefore calculated by

$$\begin{cases} J = \frac{8\sqrt{3}\,I}{9D^2} \\ \sigma = \frac{JL}{U} \end{cases} \tag{S1}$$

where D=120 μm is the typical diameter of an HC-ZnO microrod and L=1 mm is the distance between two electrodes.

SEM characterization. An HC-ZnO microrod was directly placed onto a polished highly-conductive silicon wafer for morphological examination by SEM (Hitachi SU9000).

XPS and valence band spectrum. The XPS and valence band spectra were acquired by a photoelectron spectrometer (Thermo Fisher Scientific ESCALAB 250Xi) with monochromatic Al-Kα radiation and low-energy electron flooding for charge compensation. The binding energies were calibrated using C1*s* hydrocarbon peak at 284.8 eV to compensate to surface charges. The background elimination and multi-peak fitting were performed in the equipment software (Thermo Advantage).

Temperature-dependent EL, PL and TRPL spectrum. A spectrometer (Horiba Jobin Yvon iHR550) equipped with a 100 lines/mm grating and a 5×/*NA*0.13 objective (Thorlabs LMU-5×-NUV) was employed to acquire temperature-dependent EL and PL spectra. For EL spectral analysis, the electrodes were synthesized similar with the *I*-*V* measurement as mentioned above. For PL spectra acquisition, a 325-nm-line CW He-Cd laser (Kimmon Koha IK3301R-G) was used as excitation source. For TD-TRPL measurement, an optically triggered streak camera system (Optronis SG-10) was used and the excitation source was a 325-nm-line femtosecond pulsed laser (Coherent Libra-F-HE) with a pulse duration of 150 fs. In all above-mentioned spectral measurements, the HC-ZnO microrod was horizontally placed onto a plate-heater in a vacuum chamber, in which the temperature can be controlled from 83 K to 723 K by liquid nitrogen.

Raman spectrum analysis and temperature calibration. The Raman spectra were acquired by a high-resolution spectrometer (Horiba Jobin Yvon iHR550) equipped with a 2400 lines/mm grating in backscattering configuration. A 633-nm He-Ne linear polarization laser (Thorlabs HNL210) was employed as the excitation source. In order to activate the LO modes (576 cm^{-1} and 589 cm^{-1}), the laser beam with the polarization perpendicular to the *c*-axis was focused down to ~2 μm on the surface of a horizontally placed HC-ZnO microrod by a 20×/*NA*0.40 objective (Olympus MPLN20×). It should be noted that the Raman peak at 437 cm^{-1} (E_2^{high} mode) was inactive under this configuration.

During EL emission of HC-ZnO, the temperature elevation resulting in the thermal expansion of the lattice varied the Raman shift of E_2^{high} mode (with a polarization of

excitation laser parallel to the *c*-axis). Therefore, the lattice temperature, *T*, can be determined by the Raman peak shift Δ_T, which is calculated by (Cusco *et al.*, 2007)

$$\Delta_T = -\omega_0\gamma \int_0^T [\alpha_c(T) + 2\alpha_c(T)]\, dT \qquad \text{(S2)}$$

where ω_0=437 cm^{-1}and γ=2.02 is the Grüneisen parameter of E_2^{high}; $\alpha_c(T)$ and $\alpha_a(T)$ are the linear thermal expansion coefficients of ZnO parallel and perpendicular to the *c*-axis, respectively. They are described as (Iwanaga *et al.*, 2000)

$$\begin{cases} \alpha_c(T) = 5.2042 + 0.522 \times 10^{-5}T + 12.13 \times 10^{-9}T^2 \\ \alpha_a(T) = 3.2468 + 0.623 \times 10^{-5}T + 12.94 \times 10^{-9}T^2 \end{cases} \qquad \text{(S3)}$$

Excitation probability at different ambient temperatures. For thermal ionization of a deep center, the electron had to absorb sufficient energy from the lattice. According to the Eq. (1), the influence of ambient temperature and Joule heating can be rewritten by substitution of *T* with T_p in Eq. (2). Excitation probability can therefore be derived as

$$W \sim\ exp\left\{\frac{E_T}{\hbar\omega}\left[1 - \frac{\beta}{4} - ln\left(\frac{4}{\beta}\left(exp\frac{\hbar\omega}{k_B T_{amb} exp\left(\frac{\alpha\sigma U^2}{8}\right)} - 1\right)\right)\right]\right\} \qquad \text{(S4)}$$

where E_T=2.2 eV is the thermal binding energy of electron from V_{Zn}^{2-} to CB (see Figure 4(a)).

Simplified dynamics model of electron transitions. The schematic of electron excitation and radiative recombination in an HC-ZnO microrod for EL emission is shown in Figure S1, in which the non-radiative recombination and fine energy of V_o and V_{Zn} are ignored.

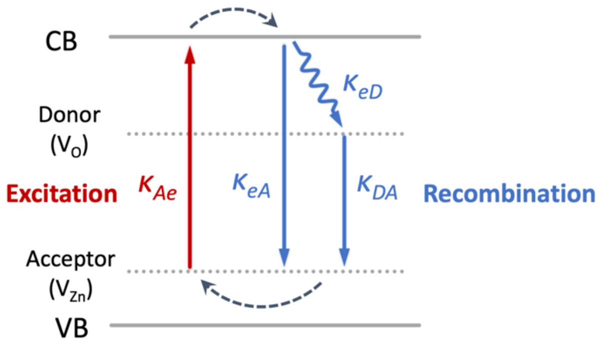

Figure S1. Schematic of electron excitation and radiative recombination in an HC-ZnO microrod for EL emission. Related to **Figure 4.**

For the simplified energy levels system, the rate equations are described as

$$\begin{cases} \frac{dn}{dt} = -\kappa_{eA}n - \kappa_{eD}n + \kappa_{Ae}n_A \\ \frac{dn_A}{dt} = -\kappa_{Ae}n_A + \kappa_{eA}n + \kappa_{DA}n_D \\ \frac{dn_D}{dt} = -\kappa_{DA}n_D + \kappa_{eD}n \end{cases} \quad \text{(S5)}$$

where n_A is the concentration of electron at the acceptor level of V_{Zn} and κ_{Ae} is the rate of excitation. For a steady-state EL emission, the dynamic equilibrium of electron transitions between CB, acceptor level and donor level are expressed as

$$\frac{dn}{dt} = \frac{dn_A}{dt} = \frac{dn_D}{dt} = 0 \quad \text{(S6)}$$

Considering Eq. (S5), $\kappa_{DA}n_D = \kappa_{eD}n$.

Chromaticity values of EL emission. The chromaticity values were calculated from EL spectra, following the standard color matching functions in CIE1931. The tristimulus values were calculated by

$$\begin{cases} X = \int_{380}^{780} I(\lambda)x'(\lambda)d\lambda \\ Y = \int_{380}^{780} I(\lambda)y'(\lambda)d\lambda \\ Z = \int_{380}^{780} I(\lambda)z'(\lambda)d\lambda \end{cases} \quad \text{(S7)}$$

where $I(\lambda)$ is the intensity distribution of an EL spectrum in the range of 380 nm to 780 nm; $x'(\lambda)$, $y'(\lambda)$ and $z'(\lambda)$ are CIE's color matching functions. The chromaticity values (x, y) in CIE1931 color space were therefore derived by

$$\begin{cases} x = \frac{X}{X+Y+Z} \\ y = \frac{Y}{X+Y+Z} \end{cases} \quad \text{(S8)}$$

SUPPLEMENTAL REFERENCES

Cusco, R., Alarcon-Llado, E., Ibanez, J., Artus, L., Jimenez, J., Wang, B.G., and Callahan, M.J. (2007). Temperature dependence of raman scattering in ZnO. Phys. Rev. B *75*, 11.

Iwanaga, H., Kunishige, A., and Takeuchi, S. (2000). Anisotropic thermal expansion in wurtzite-type crystals. J. Mater. Sci. *35*, 2451-2454.
